# Spatio-Temporal Variations in Groundwater Revealed by GRACE and Its Driving Factors in the Huang-Huai-Hai Plain, China

**DOI:** 10.3390/s20030922

**Published:** 2020-02-10

**Authors:** Youzhe Su, Bin Guo, Ziteng Zhou, Yulong Zhong, Leilei Min

**Affiliations:** 1 Key Laboratory of Geomatics and Digital Technology of Shandong Province, Shandong University of Science and Technology, Qingdao 266590, China; syz3528@163.com (Y.S.); 13646428022@163.com (Z.Z.); 2 College of Geomatics, Shandong University of Science and Technology, Qingdao 266590, China; 3 School of Geography and Information Engineering, China University of Geosciences (Wuhan), Wuhan 430078, China; zhongyl@cug.edu.cn; 4 Key Laboratory of Agricultural Water Resources, Center for Agricultural Resources Research, Institute of Genetics and Developmental Biology, Chinese Academy of Sciences, Shijiazhuang 050021, China; llmin@sjziam.ac.cn

**Keywords:** GRACE, groundwater, Huang-Huai-Hai Plain, hydrological model, water footprint

## Abstract

The Huang-Huai-Hai (3H) Plain is the major crop-producing region in China. Due to the long-term overexploitation of groundwater for irrigation, the groundwater funnel is constantly expanding and the scarcity of water resources is prominent in this region. In this study, Gravity Recovery and Climate Experiment (GRACE) and hydrological models were used to estimate the spatial-temporal changes of groundwater storage (GWS) and the driving factors of GWS variations were discussed in the 3H Plain. The results showed that GRACE-based GWS was depleted at a rate of −1.14 ± 0.89 cm/y in the 3H Plain during 2003 to 2015. The maximum negative anomaly occurred in spring due to agricultural irrigation activities. Spatially, the loss of GWS in the Haihe River Basin is more serious than that in the Huaihe River Basin, presenting a decreasing trend from south to north. Conversely, the blue water footprint (WF_blue_) of wheat exhibited an increasing trend from south to north. During the drought years of 2006, 2013, and 2014, more groundwater was extracted to offset the surface water shortage, leading to an accelerated decline in GWS. This study demonstrated that GWS depletion in the 3H Plain is well explained by reduced precipitation and groundwater abstraction due to anthropogenic irrigation activities.

## 1. Introduction

Groundwater, as an important body of water on Earth, plays a key role in global hydrological and biogeochemical cycles [1,2]. With its stable water supply conditions and good water quality, groundwater is an important source of fresh water for agricultural irrigation, industry, and urban living in many parts of the world, especially in semi-arid regions and densely populated countries [3,4]. Globally, over two billion people regard aquifers as their primary source of drinking water [5]. Furthermore, the consumption of underground water sources for irrigation used to supply the growing demand of the world’s food is estimated to be 545 km^3^/a, accounting for 43% of the total irrigation water [3]. Groundwater is being pumped at a far greater rate than it can be naturally replenished, so that the decrease in water level and the loss of groundwater storage are sustained, which have already caused a groundwater funnel in several regions and some geological environment problems, such as seawater intrusion and land subsidence [5,6].

Limited by available well networks, the conventional water level records from monitoring wells have difficulty in producing large-scale dynamic observations and evaluations. In addition, due to the lack of consistent detailed hydrogeologic information, most global-scale hydrological models do not include a groundwater flow component [7]. Alternatively, some geodetic methods can remotely monitor GWS change and land surface deformation, such as Gravity Recovery and Climate Experiment (GRACE), global positioning system (GPS), and interferometric synthetic aperture radar (InSAR) [8].

Since being launched in March 2002, GRACE has provided precise measurements of mass change by tracking variations in the Earth’s gravity field, which can be used to monitor the terrestrial part of the hydrological cycle at a spatial resolution larger than ~300 km [9]. GRACE-derived total terrestrial water storage (TWS) changes represent integrated variations in all forms of water storage above and below the surface of the Earth [10]. Therefore, it can be used to estimate GWS changes when other TWS components from soil moisture, snow/ice, and surface water are subtracted. Over the past decade, GRACE data have been used to evaluate large-scale GWS changes in many parts of the world. For instance, Rodell et al. [11] found that GRACE-derived GWS corresponded well with estimates based on monitoring well observations in the Mississippi River Basin and its four sub-basins. This was further demonstrated by studies performed in India [12,13], California’s Central Valley, USA [14,15], the Middle East [16], and the North China Plain (NCP) [17]. Admittedly, GRACE has been a powerful tool in estimating groundwater depletion rates. 

In order to better understand and quantify the impact of human activities on water resources and the water-related environment, Hoekstra et al. [18] put forward the concept of “water footprint (WF)” on the basis of virtual water theory in 2002. It was first proposed in the field of agricultural production to express the amount of water demand in the production process of agricultural products and then expanded to the amount of water needed for the production of goods and services. The WF has three components: green WF (WF_green_) is the amount of water resources stored in soil and rainwater evaporated during crop growth; blue WF (WF_blue_) is the amount of water resources used by fresh water bodies (surface water and groundwater), mainly including evaporation of irrigation water; and grey WF (WF_grey_) refers to the amount of fresh water needed to absorb the pollutant load generated in the production process of products [19]. Chen et al. [20] calculated the virtual water content of wheat and maize and their growth and production water footprints in the NCP (Hebei, Beijing and Tianjin) of China and found that blue water dominated the growth period of wheat, accounting for about 60% of the total WF of wheat. In addition, they also proposed that the total WF of maize and wheat was about 2.2-times that of the local water resources, which emphasized again that there was a contradiction between supply and demand of water resources in the NCP. Considering the serious water shortage during the wheat growing period, it is quite significant to assess the WF_blue_ of wheat in the Huang-Huai-Hai Plain (3H Plain) for the development of sustainable agriculture and the adjustment of aquatic ecosystem.

However, previous studies have paid little attention to the effect of the variation in agricultural water demand on GWS in the 3H Plain. Thus, the main objectives of this study were to: (1) characterize the spatial-temporal variations of GWS in the 3H Plain; (2) analyze the impacts of WF_blue_ of wheat, precipitation, and evapotranspiration on GWS variations. The remainder of the paper is organized as follows: Section 2 describes the study area, data collection, and processing. Section 3 focuses on the results of GWS trends and the influences of agricultural water demand, precipitation, and Palmer Drought Severity Index (PDSI). Finally, the discussion and conclusion are delivered in Section 4 and Section 5, respectively.

## 2. Data and Methodology

### 2.1. Study Area

**Figure 1:**
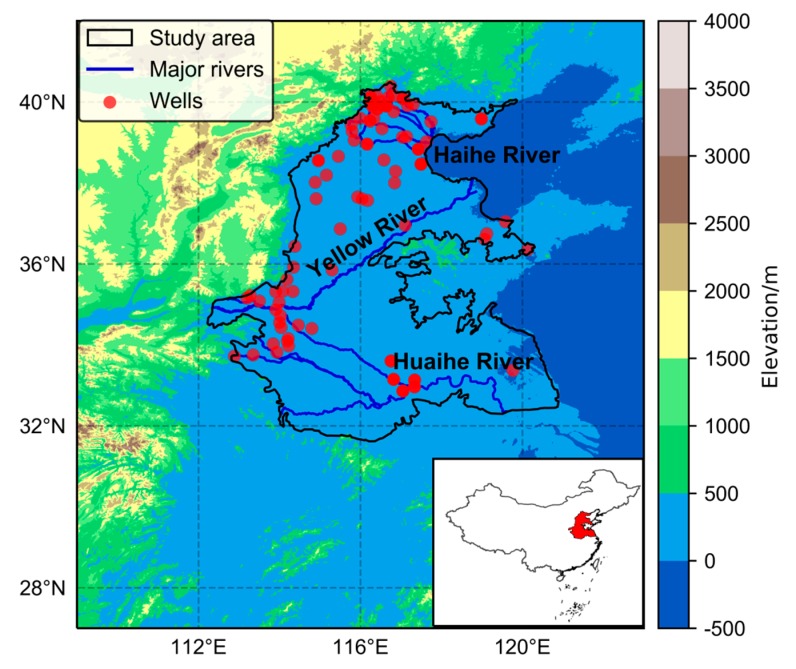
Study area of the Huang-Huai-Hai Plain (3H Plain, surrounded by the black curve) and the distribution of groundwater monitoring wells (red dots). The main rivers are shown in blue. The insert map shows the location of the 3H Plain in China.

The 3H Plain is the second largest plain in China and one of the largest aquifer systems in the world. Confined within 31°36′–40°29′ N and 112°13′–120°53′ E, the 3H Plain covers an area of ~300,000 km^2^, spanning seven provinces and cities of Beijing, Tianjin, Hebei, Shandong, Henan, Anhui, and Jiangsu (Figure 1). There are three main rivers distributed in this region, including the Haihe River, the Yellow River, and the Huaihe River, from north to south, respectively. The lower reaches of the Yellow River run naturally across the central part of the plain, which divide the region into two parts: the Haihe Plain in the north and the Huanghuai Plain in the south. As an important agricultural region, the 3H Plain plays a substantial role in guaranteeing the food security of China, with 35,000 km^2^ of highly-intensive arable land, accounting for 19% of the country’s crop production area [21]. Here, the annual double-cropping system of winter wheat and summer maize is the most popular planting pattern. In addition, the yield of wheat and maize accounts for about 61% and 31% of the total national output, respectively [22]. The average annual precipitation is about 600–900 mm. Due to the continental monsoon climate, precipitation mainly occurs in summer (June to August), with less precipitation in spring (March to May). Therefore, winter wheat suffers from serious water shortages during the filling period, with only 25%~40% of the water requirement coming from precipitation and more than 60% from groundwater [23]. Additionally, rapid industrialization and urbanization, coupled with the increase in temperature and changing precipitation patterns, has resulted in the rapid growth of regional water demands [24]. Meanwhile, overexploitation of groundwater has led to a decrease in the groundwater level at a rate of 1 m per year and a large area of depression cones in the past two decades [25,26].

### 2.2. Data Collection and Processing

GRACE-derived TWS comprises all forms of water stored in the land, i.e., surface water, snow, soil moisture, and groundwater. Thus, GWS anomalies (GWSA) can be isolated by removing surface water reservoir storage anomalies (RESSA), soil moisture storage anomalies (SMSA), and snow water equivalent storage anomalies (SWESA) [15]:

(1) GWSA=TWSA−RESSA−SMSA−SWESA,

where SWESA and SMSA are estimated using the four land surface models (LSMs) of the Global Land Data Assimilation System (GLDAS). Based on the statistics of surface water reservoir storages from the China Water Resources Bulletins (CWRB), published by the Ministry of Water Resources of China (MWR) [27], the RESSA trend from GRACE-based TWSA during 2003 to 2015 was removed, whereas the contribution of RESSA was very small (~0.3 km^3^/a). The groundwater table changes from the monitoring wells and the WaterGAP Global Hydrological Model (WGHM) were utilized to verify the accuracy of GWSA from GRACE. Precipitation from Tropical Rainfall Measuring Mission (TRMM) 3B43V7, evapotranspiration from GLDAS, drought index, and WF_blue_ of wheat were used to evaluate the effect of climate change and anthropogenic irrigation activities on groundwater resources. Table 1 summaries the data used in this study.

#### 2.2.1. TWSA from GRACE

TWSA can be derived from the GRACE data, which are available in two forms: spherical harmonics (SH) data and mass concentration (mascon) solutions. Compared to the SH solutions, the GRACE mascon solutions have some pivotal benefits. As to mascon, the noise from GRACE observations at the Level-2 processing step is easily filtered out by implementing geophysical constraints. Thus, mascon solutions better deal with the mass leakage problem relative to the SH solutions, which are beneficial for studies of the regional mass change [8]. Considering these reasons, GRACE mascon solution from Jet Propulsion Laboratory (JPL) was adopted in this study. We used the latest JPL mascon RL06 datasets with a 0.5-degree spatial resolution. The grid values needed to be multiplied by the corresponding scale factor to recover the leakage signals. The scaling factors were provided by the JPL website (https://grace.jpl.nasa.gov/opendap/allData/tellus/L3/mascon/RL06/JPL/). Given that mascon data were anomalies relative to the 2004 to 2009 time-mean baseline, the base period of other anomalies data was the same in this study. The missing data in GRACE were obtained by linear interpolation. 

#### 2.2.2. SWESA, SMSA, and Evapotranspiration from GLDAS

Developed by the National Aeronautics and Space Administration (NASA) and the National Oceanic and Atmospheric Administration (NOAA), the GLDAS LSMs can simulate optical fields of land surface states and fluxes [28] and are widely used in presenting global surface water variations [29]. We used the average SMSA and SWESA from four versions of LSMs provided by GLDAS, i.e., National Centers for Environmental Prediction/Oregon State University/Air Force/Hydrologic Research Lab (Noah) [30], Common Land Model (CLM) [31], MOSAIC [32], and Variable Infiltration Capacity (VIC) [33], with their respective number of soil moisture layers being 4, 10, 3, and 3, and corresponding depths reaching 2.0 m, 3.4 m, 3.5 m, and 1.9 m. Similarly, evapotranspiration was also obtained from the average of four GLDAS LSMs. Notably, Pan et al. [34] found that the GLDAS-based evapotranspiration had a visible underestimation of about 60 mm/y in the dry season (March–May). Thus, we considered increasing the monthly evapotranspiration by 20 mm in the dry season.

#### 2.2.3. GWSA from Monitoring Wells

The groundwater level data from 100 monitoring wells were collected from the China Institute of Geological Environment Monitoring (CIGEM) during 2003 to 2013 [35]. Some raw well observation statistics had serious quality problems with abnormal jumps, data gaps, and outliers, which were removed in the preprocessing stage. The GWSA from monitoring wells is calculated as follows [36,37]:

(2) $$GWSA=\sum_{i}^{N}S_{j}W_{j}\Delta h_{j}/\sum_{j}^{N}W_{j},$$

where *S_j_* is the specific yield for the unconfined aquifers or storativity for confined aquifers; *N* refers to the number of grid cells divided in the study area; *W_j_* is the weight of each grid cell (the cosine of the latitude of the corresponding cell); and Δ*h_j_* is the mean of the well water level variations in each grid cell. We segmented the study area into a 1 by 1 degree mesh where each cell of the mesh was assigned the mean value of all wells within it [38]. For the 3H Plain, a mean specific yield value of 0.06 [8,39] was used to transform well water level variations to GWS variations.

#### 2.2.4. GWSA from WGHM

WGHM 2.2d, provided by the University of Frankfurt (FRA), is one of the hydrological water balance models (HMs), which was developed to evaluate global water availability and water uses. It simulates continental water flows among all relevant water storage compartments, including groundwater and surface water abstractions [40]. Therefore, we could estimate GWS variations based on these data.

#### 2.2.5. Precipitation and Drought Index

In this study, the Tropical Rainfall Measuring Mission (TRMM) 3B43V7 precipitation product was used to obtain monthly precipitation in the 3H Plain. The TRMM is a result of a cooperative mission between NASA and the Japan Aerospace Exploration Agency (JAXA), providing precipitation data between 50° S and 50° N [41].

The Palmer Drought Severity Index (PDSI) was used to assess the dry and wet conditions in our study. Different from many other drought indices that are based on precipitation alone, the PDSI uses both precipitation and surface air temperature as inputs, which allows it to interpret the fundamental effect of surface warming on dry and wet periods [42]. The values of PDSI usually range from −4 to 4, and when the value is greater than 0, it indicates a wet state. Conversely, it is considered to be drought when the PDSI is smaller than 0. The different values reflect different drought or wet levels. 

#### 2.2.6. WF_blue_ of Wheat

Due to the uneven seasonal distribution of precipitation, winter wheat suffers from a serious water shortage during its growing period compared with summer maize and, thus, the production of winter wheat relies mainly on supplementary irrigation by exploiting groundwater in the 3H Plain [43]. Therefore, the WF_blue_ of wheat obtained from Zhuo et al. [44] was used to illustrate the spatial change in agricultural irrigation of winter wheat and its impact on GWS so as to provide a basis for rational utilization of groundwater and improvement of agricultural irrigation efficiency.

### 2.3. Uncertainty Assessment

The TWSA uncertainty estimation was based on the method proposed by Landerer and Swenson [45] and Scanlon et al. [46]. First, the linear trends and seasonal components were removed from TWSA to obtain the residuals. Then, given that the interannual signals also contributed greatly to the TWSA in the 3H Plain, we further removed it by fitting a 13-month moving average to the residuals. The root mean square (RMS) of the residuals approximates the measurement uncertainty in TWSA. For SMSA+SWESA, the uncertainty was estimated from the monthly standard deviation (STD) among the four GLDAS models [47,48]. Considering the error propagation law during the least squares fit and uncertainties in TWSA and SMSA+SWESA [49], we estimated the uncertainty in the GWS trend.

## 3. Results

### 3.1. Temporal Changes in GWSA

**Figure 2:**
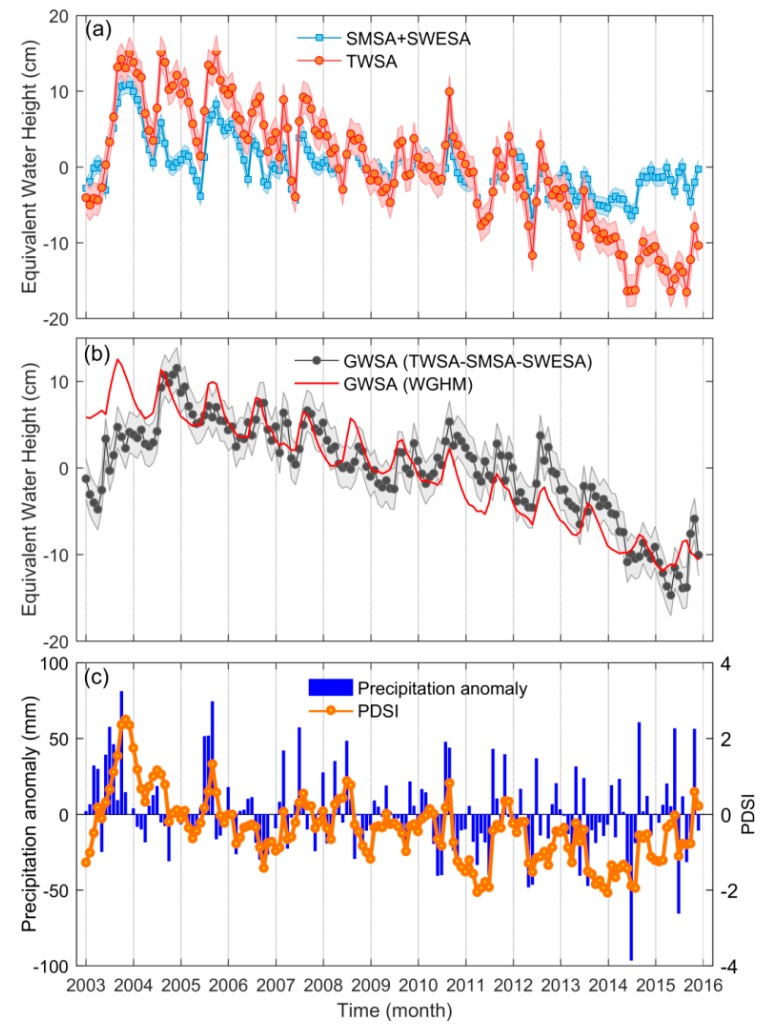
(**a**) Monthly GRACE-based terrestrial water storage anomalies (TWSA) and soil moisture storage anomalies (SMSA)+snow water equivalent storage anomalies (SWESA) derived from GLDAS. The blue and red shaded areas represent the uncertainties in TWSA (2.06 cm) and SMSA+SWESA (1.15 cm); (**b**) monthly GWSA derived from GRACE and WGHM. The shaded area represents the uncertainties in GWSA (2.37 cm); and (**c**) monthly precipitation anomalies and PDSI.

Figure 2a shows the temporal changes of TWSA and SMSA+SWESA in the 3H Plain from 2003 to 2015. The monthly TWSA exhibited a decreasing trend from 2003 to 2015, with a decline rate of −1.73 ± 0.20 cm/y, and the SMSA+SWESA decreased at a rate of −0.59 ± 0.12 cm/y. Obviously, the SMSA+SWESA presented a smaller amplitude than TWSA, while their phases coincided relatively well. The TWSA change rate stayed relatively stable before September 2010 (−1.08 ± 0.04 cm/y) but more than doubled that from September 2010 to December 2015 (−3.08 ± 0.05 cm/y), which naturally divided the trend curve into two sub-periods. 

Figure 2b shows the temporal changes of GWSA based on GRACE and WGHM in the 3H Plain during 2003 to 2015. Similar with the TWSA, GWSA derived from GRACE also showed a decreasing trend, with a rate of −1.14 ± 0.89 cm/y. The change rates of GWSA were estimated to be −0.50 ± 0.31 cm/y and −2.95 ± 0.39 cm/y for before and after September 2010, respectively, indicating that the decline in GWS accelerated after September 2010. GWSA derived from the WGHM declined at a rate of −1.61 ± 0.08 cm/y during 2003 to 2015, significantly higher than that of the GRACE-based GWSA during the same periods. Döll et al. [50] and Feng et al. [39] found that WGHM 2.2a and WGHM 2.2b overestimated the GWS in the NCP, respectively. In our study, there still exists an overestimation in the latest WGHM 2.2d model compared with GRACE in the 3H Plain, whereas this model improved the simulation of GWS depletion.

Figure 2c shows the temporal changes of monthly precipitation anomalies and PDSI in the 3H Plain from 2003 to 2015. Overall, the change in PDSI was consistent with precipitation anomalies and GWSA on a monthly scale. Particularly, the values of PDSI are usually negative in the 3H Plain since September 2010, suggesting a drought condition during this period, which also agrees with the finding that the GWS had an accelerated decrease rate after September 2010. The precipitation was 289.2 mm higher in 2003 than the 13-year average of 837.3 mm and GWSA also presented a rapid rebound during this period. Moreover, during October 2010 to July 2011, the continuous decline of GWSA was mainly in response to the nine-month negative anomalies of precipitation.

### 3.2. Intercomparison Analysis of GWSA from GRACE and Monitoring Wells

**Figure 3:**
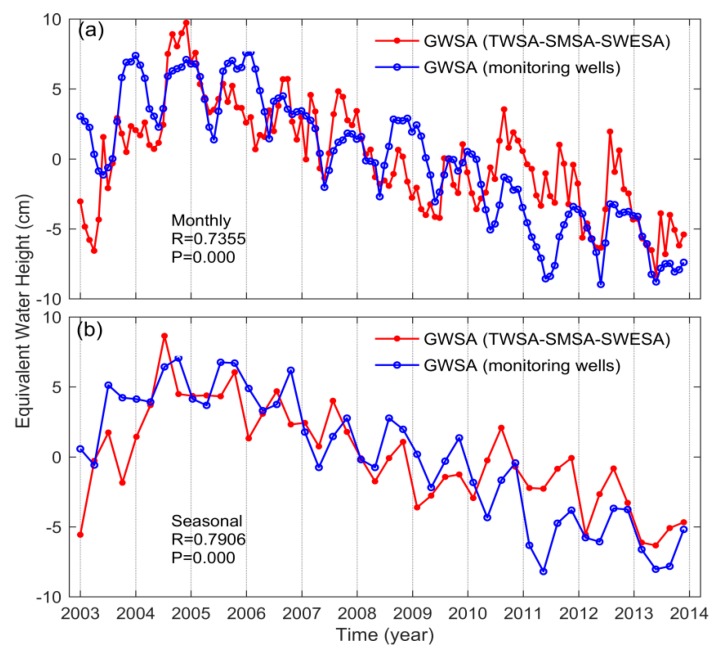
(**a**) Monthly and (**b**) seasonal variations between GRACE-derived GWSA and monitoring well observations in the 3H Plain.

**Figure 4:**
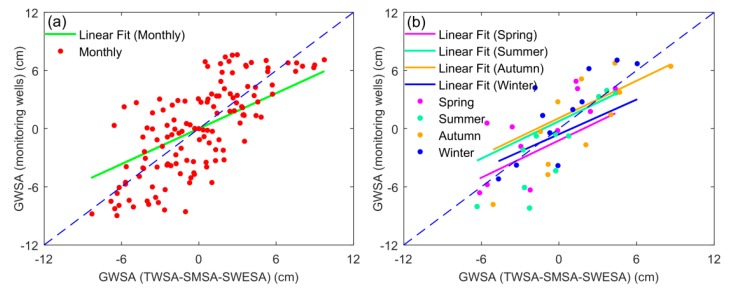
Comparisons of (**a**) monthly and (**b**) seasonal GWSA between GRACE and monitoring well observations in the 3H Plain.

The monthly and seasonal GWSAs play a crucial role in the study of the hydrological cycle. In this study, the four seasons refer to spring (March–May), summer (June–August), autumn (September–November), and winter (December–February). We compared the monthly and seasonal GRACE-based GWSA with the monitoring well observations from 2003 to 2013, as seen in Figure 3 and Figure 4. Obviously, both GRACE and monitoring well data present significant groundwater depletion in the 3H Plain. In terms of monthly scale, the correlation of GWSA between GRACE and monitoring wells reached 0.74 and the root-mean-square error (RMSE) was 2.98 cm. For seasonal scale, the correlation between the two sequences was 0.79 and the RMSE was 1.58 cm. In addition, GWSA from GRACE and monitoring wells was calculated for each season separately (Figure 4b). We found that the correlation between GWSA from GRACE and monitoring wells for the four seasons, from 2003 to 2013, is summer (0.88) > autumn (0.82) > winter (0.81) > spring (0.76), and the fitting lines are all close to a 1:1 line. On the whole, the monthly and seasonal GWSA derived from GRACE and monitoring wells compared favorably in the aspect of seasonal peaks and phases, which proves that it is feasible to study GWS changes using GRACE in the 3H Plain [51,52]. However, there are still differences between GWSA derived from GRACE and monitoring wells. For example, GRACE detected a lower GWSA from 2003 to early-2004, disagreeing with the monitoring well records in terms of amplitude. Additionally, GWSA derived from GRACE was much larger than that from monitoring wells during September 2010 to December 2011. Given the inadequate spatial resolutions due to limited available monitoring well networks, the monitoring wells fail to reflect the holistic situation in some particular cases.

### 3.3. Spatial Distribution in GWSA and WF_blue_ Anomalies of Wheat

**Figure 5:**
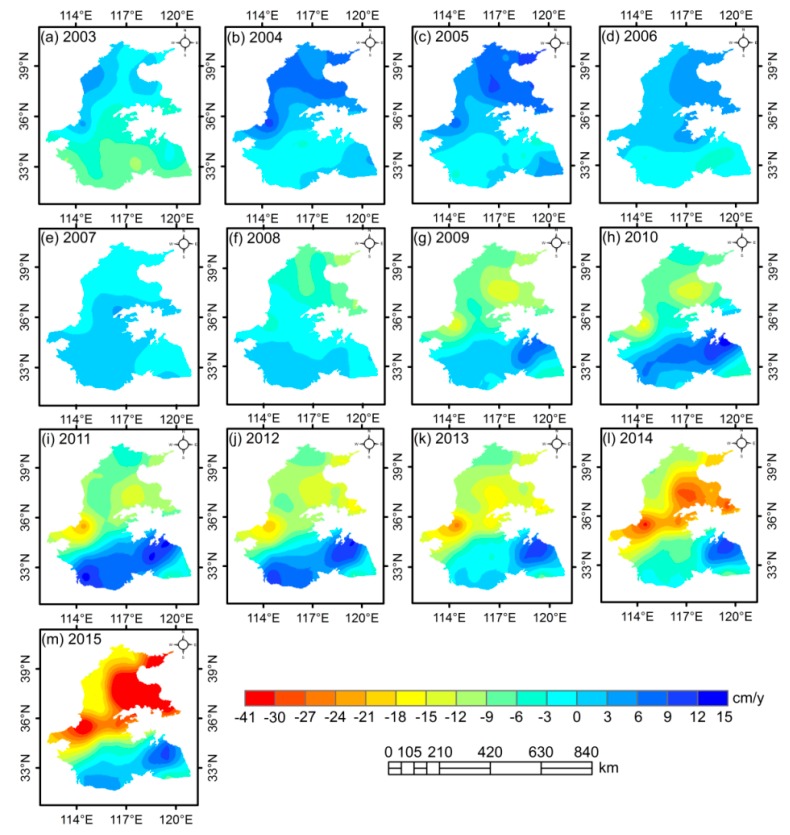
Spatial distribution of GWSA based on GRACE in the 3H Plain from 2003 to 2015.

Figure 5 shows the spatial distribution of annual GWSA derived from GRACE in the 3H Plain from 2003 to 2015. Overall, the annual GWSA experienced a marked decreasing trend throughout all of the study period, whereas the change was quite volatile in some areas. Compared with 2003, the GWSA rebounded significantly in 2004, which is in good agreement with the temporal changes seen in Figure 2b. In addition, GWSA was negative in the Haihe River Basin from 2007 to 2015, indicating that these regions suffered serious groundwater loss. However, the GWSA presented a fluctuating rising trend in the Huaihe River Basin for the period of 2003 to 2012 and it increased greatly during 2010 to 2012, which can be verified by three small peaks for the same period in Figure 2b.

**Figure 6:**
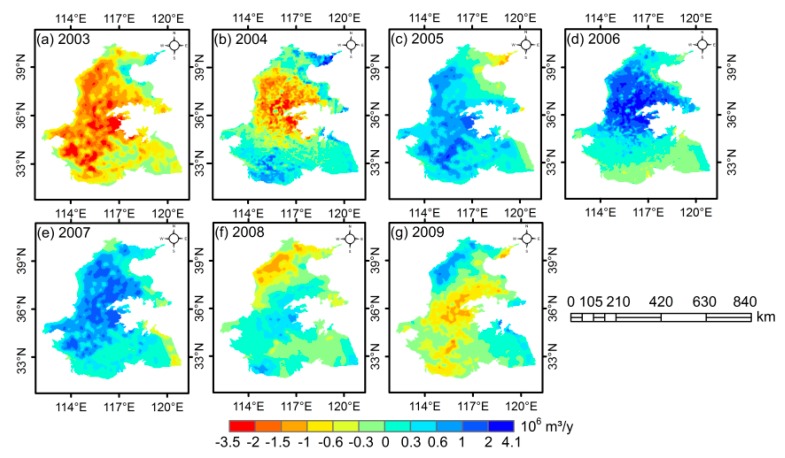
Spatial distribution of WF_blue_ anomalies of wheat in the 3H Plain from 2003 to 2009.

Agricultural irrigation is an important factor affecting GWSA variations in the 3H Plain. There were significant spatial differences in the annual WF_blue_ anomalies of wheat during 2003 to 2009 (Figure 6). The WF_blue_ anomalies of wheat changed prominently in most regions of the Haihe River Basin, with the lowest anomalies of −3.5 × 10^6^ m^3^ in 2003 and the peak value of 4.1 × 10^6^ m^3^ in 2006. Overall, the annual WF_blue_ anomalies of wheat experienced an increasing trend from 2003 to 2007, whereas there has been a significant decline since 2008. In addition, WF_blue_ anomalies of wheat were obviously lower in a rainy year (2003) compared with that in a drought year (2006), illustrating that precipitation influences GWS by altering groundwater recharge and groundwater use [53,54].

## 4. Discussion

### 4.1. Effects of Precipitation and Evaporatranspiration on GWS

**Figure 7:**
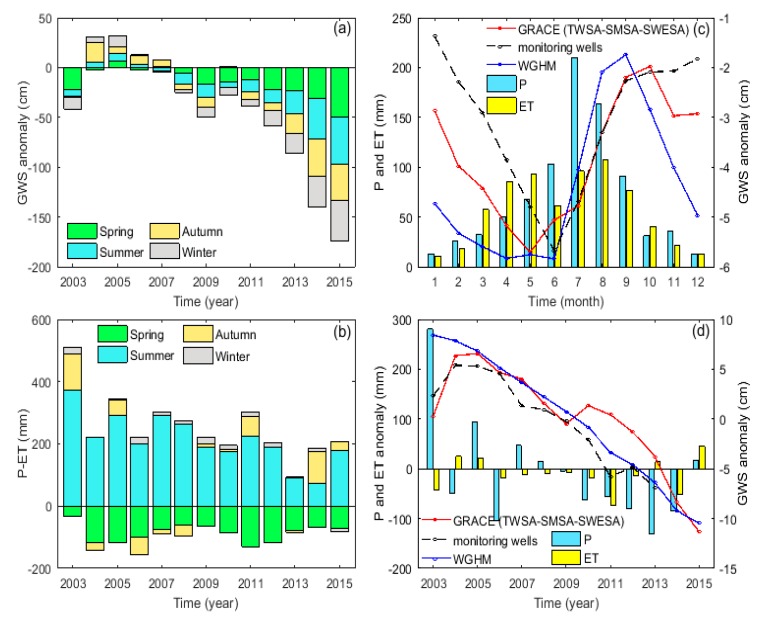
Temporal variations of GWSA in the 3H Plain during 2003 to 2015: (**a**) seasonal GWSA variations derived from GRACE; (**b**) seasonal P-ET (precipitation-evapotranspiration); (**c**) intra-annual changes in monthly GWSA derived from GRACE, monitoring wells, and WGHM. The blue and yellow bars represent the monthly average precipitation and evapotranspiration, respectively; and (**d**) inter-annual changes in GWSA derived from GRACE, monitoring wells, and WGHM. The blue and yellow bars represent the annual anomalies of precipitation and evapotranspiration, respectively.

Precipitation and evapotranspiration undoubtedly have the greatest impact on GWS of all the natural factors. In a region, precipitation is the input of water, while evapotranspiration is the output of water. Thus, precipitation minus evapotranspiration (P-ET) is regarded as the net recharge of surface and groundwater [55]. Figure 7a,b shows the seasonal differences of GRACE-derived GWSA and P-ET during the period of 2003 to 2015, respectively. The maximum negative GRACE-based GWSA occurred in spring (−153 mm), then in summer (−131 mm). On the contrary, P-ET reached its maximum in summer (242 mm) and its minimum in spring (−89 mm). Obviously, the principal natural factors for the rapid decline of GWS in spring are less precipitation and intensive evapotranspiration. Considering that the largest P-ET occurred in the summer during 2003 to 2015, so the rapid decline of GWS in this period was mostly driven by human activities. In general, GWSA was negative in four seasons after 2007 and presents a downward trend year-by-year, which further indicates that GWS is not only affected by precipitation and evapotranspiration, but also by agricultural distribution and human activities [56]. 

There were also clear differences between GWSA and precipitation (evapotranspiration) on an intra-annual scale (Figure 7c). The annual average precipitation is 846 mm, about 57% of which occurs in summer and 18% in spring. Different from precipitation, evapotranspiration distributes asymmetrically in the four seasons, characterizing an M shape annually, with spring and summer accounting for 34.6% and 38.9% respectively [57]. P-ET reaches the minimum in spring, which makes the groundwater decrease rapidly due to the large water demand of winter wheat during this period. On the contrary, the rebound of GWS in July and August is closely related to the rainy season in the 3H Plain. Particularly, the maximum GWSA occurs in September or October, while the maximum P-ET is found in July, proving that groundwater has a delayed response to precipitation for nearly 2–3 months because of the slow water infiltration [58,59,60]. In addition, corresponding to PDSI in the same period, annual precipitation anomalies were negative during 2010 to 2014 (Figure 7d), indicating that this period was in a relatively drought condition, which is in good agreement with the long-term GWS depletion signal inferred from GRACE.

### 4.2. Effects of Anthropogenic Irrigation Activities on GWS

In the 3H Plain, it is widely recognized that winter wheat is sown at the beginning of October and harvested in June of the second year, and that summer maize is then sown immediately afterwards and harvested at the end of September [61]. Yang et al. [21] estimated that the average seasonal evapotranspiration of summer maize and winter wheat is 354.8 mm and 521.5 mm, respectively, in the 3H Plain and further found that a high-evapotranspiration belt of wheat was located in the middle part of the 3H Plain. Their estimate also agrees favorably with the spatial variations of the WF_blue_ anomalies of wheat in this study. For winter wheat, the average precipitation is less than 300 mm during the growth period. However, the water requirement of winter wheat is about 400 to 550 mm during the whole growth period and the coupled rain degree for the winter wheat growing season is only 0.4 [62]. Furthermore, the peak period of water consumption of winter wheat is the filling period (April and May), accounting for 57% of its total water consumption [63]. Nevertheless, the average precipitation is less than 150 mm during this period [64], which further exacerbates the declines in TWS and GWS in the spring. Therefore, winter wheat is considered to be the largest contributor to the GWS decline in the cropping system [65].

**Figure 8:**
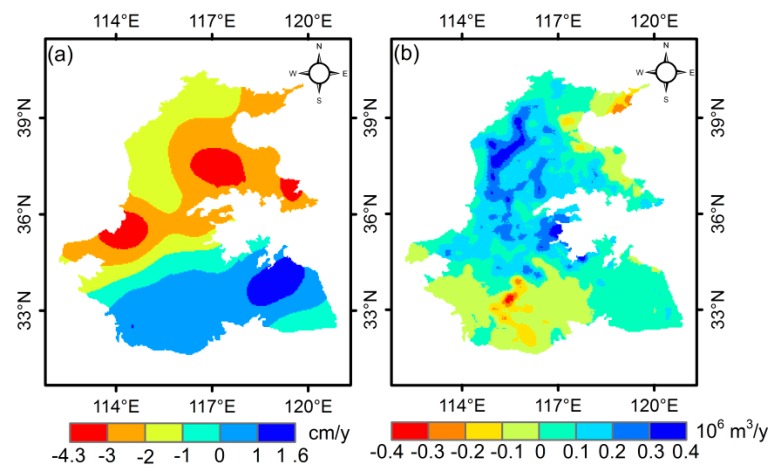
Change rates of (**a**) GRACE-derived GWSA from 2003 to 2015 and (**b**) WF_blue_ anomalies of wheat from 2003 to 2009 in the 3H Plain.

Figure 8 presents the long-term change rates of GRACE-based GWSA and the WF_blue_ anomalies of wheat. GWSA exhibited a declining trend of −2.33 ± 0.18 cm/y in the Haihe River Basin from 2003 to 2015, while most regions in the Huaihe River Basin showed an increasing trend of 0–1 cm/y (Figure 8a). Accordingly, the WF_blue_ anomalies of wheat presented an increasing rate (0 to 0.4 × 10^6^ m^3^/y) in most regions, whereas there was a remarkable decline in the Midwest of the Huaihe River Basin during 2003 to 2009 (Figure 8b). The largest rising rate (0.2 × 10^6^–0.4 × 10^6^ m^3^/y) of the WF_blue_ anomalies of wheat mostly occurred in the piedmont plain area, which is a high-intensity agricultural irrigation area as well as a main shallow groundwater exploitation region [66,67]. The overall trends of GWSA and WF_blue_ anomalies of wheat agreed well, which demonstrates that the increasing agricultural irrigation has a primary effect on GWS withdrawal in the 3H Plain [68].

### 4.3. Comparison with Other Studies

Feng et al. [68] combined GRACE Release-05 (RL05) SH solution generated by the University of Texas at Austin’s Center for Space Research (CSR) with GLDAS data from 2003 to 2010 in the NCP and found that GWS depleted at a rate of −2.2 ± 0.3 cm/y in terms of equivalent water height (EWH). Zhao et al. [8] evaluated the GWS trend in the NCP using three mascon RL05 solutions, monitoring well data, and global positioning system (GPS) sites, concluding that GWS depleted at a rate of −1.7 ± 0.1 cm/y from 2004 to mid-2016 and accelerated to −3.8 ± 0.1 cm/y from mid-2013 to mid-2016. According to the bulletin statistics (2003 to 2012) of groundwater decline in the Haihe River Basin, the annual mean deep groundwater consumption was nearly 6.3 km^3^/y [69]. Moreover, at least 60% (−3.8 km^3^/y, i.e., −4.42 cm/y in EWH) of the deep groundwater withdrawal within the Haihe River Basin occurred in the eastern central plain (ECP), which is close to our result, with a declining rate of −3 to −4.3 cm/y (Figure 8a).

In this study, a decreasing rate of −2.33 ± 0.18 cm/y was estimated in the Haihe Plain from 2003 to 2015 using the latest mascon JPL RL06, which is consistent with the results of Feng et al. [68] and Zhao et al. [8]. In the 3H Plain, GRACE detected GWS depletion rates of 0.5 ± 0.31 cm/y from January 2003 to September 2010 and −2.95 ± 0.39 cm/y from October 2010 to December 2015. The average mining modules in the Huaihe River Basin are below 3 × 10^4^ m^3^/(km^2^·y) and only 31% of the total groundwater resources are exploited, while it is above 7 × 10^4^ m^3^/(km^2^·y) in the Haihe River Basin [70]. Therefore, more groundwater is exploited in the Haihe Plain, leading to the groundwater depletion rate appearing as an upward trend from south to north in the 3H Plain.

## 5. Conclusions

In this study, we combined Gravity Recovery and Climate Experiment (GRACE) and hydrological models to evaluate the spatial-temporal distributions of groundwater storage (GWS) and analyze the influence of recent climate change and anthropogenic irrigation activities on GWS variations in the 3H Plain. The main conclusions were as follows:

(1) GWSA was estimated using GRACE, monitoring wells, and WGHM. GRACE detected a GWS depletion rate of −1.14 ± 0.89 cm/y during 2003 to 2015. The GWS change rates stayed relatively stable before September 2010 and then began an accelerated decline. For monitoring well observations, the GWS depletion rate was −1.23 ± 0.09 cm/y from 2003 to 2013. The GWS depletion rate from WGHM (−1.61 ± 0.08 cm/y) was remarkably higher than that from GRACE and the monitoring wells.
(2) In terms of spatial changes, GWS presented a decreasing trend from south to north, but the WF_blue_ of wheat was the opposite, confirming that a considerable proportion of irrigation water comes from groundwater and contributes to groundwater overdraft.
(3) The correlation coefficients between GRACE and the monitoring wells reached 0.74 on the monthly scale and 0.79 on the seasonal scale from 2003 to 2013. Therefore, it is feasible to use GRACE data to monitor GWS variations in the 3H Plain.
(4) On the intra-annual scale, P-ET reached a minimum in spring (March-May), which made the groundwater decrease rapidly due to the large amount of irrigation of winter wheat during this period. However, the rebound of GWS in July and August was driven by more precipitation in the summer.
(5) Similar to temporal variability in PDSI, annual precipitation anomalies have been negative during 2010 to 2014. This continuous drought condition agrees well with the long-term GWS depletion signal inferred from GRACE and WGHM.

In conclusion, the uneven spatial-temporal distribution of precipitation, coupled with intense seasonal evapotranspiration and persistent groundwater exploitation, lead to the long-term GWS depletion in the 3H Plain. However, due to the large uncertainties and coarse resolution, GRACE is limited in groundwater hydrology. InSAR provides an alternative to monitor high resolution land subsidence due to GWS changes with a millimeter-level accuracy. Thus, we will use InSAR technology to monitor the land deformation of the 3H Plain in future research work.

## Figures and Tables

**Table 1:** Summary of the data used in this study.

Data Source/Type	Data Name	Data Access	Spatial and Temporal Resolution, Coverage Period
GRACE	TWSA	http://grace.jpl.nasa.gov/	1° × 1°, monthly, 2003–2015
GLDAS-LSMs	SMSA+SWESA, Evapotranspiration	https://disc.gsfc.nasa.gov/	1° × 1°, monthly, 2003–2015
MWR	RESSA	CWRB	Region sum, annual, 2003–2015
Monitoring Wells	GWS	CIGEM	100 wells, monthly, 2003–2013
WGHM	GWS	FRA	0.5° × 0.5°, monthly, 2003–2015
TRMM 3B43V7	Precipitation	https://pmm.nasa.gov/data access/downloads/trmm/	0.25° × 0.25°, monthly, 2003–2015
Drought Index	PDSI	http://climexp.knmi.nl/	0.5° × 0.5°, monthly, 2003-2015
Agricultural WF	WF_blue_ of Wheat	https://waterfootprint.org/	5′ × 5′, annual, 2003–2009
